# Supplementary material for: Predation and fragmentation portrayed in the statistical structure of prey time series
Source: BMC Ecol. 2009 May 6;9:10. doi: 10.1186/1472-6785-9-10 (PMC2689204; doi:10.1186/1472-6785-9-10)
Supplement: Additional file 2 — Voles and related classes ODDox Documentation. ODDox documentation of the agent-based model (ALMaSS) applied by Hendrichsen et al. The documentation is started by activating main.html. [file 1472-6785-9-10-S2.zip › Vole_ODDox/functions_func_0x73.html]

ALMaSS ODDox: Class Members - Functions

- Main Page
- Related Pages
- Classes
- Files

- Alphabetical List
- Class List
- Class Hierarchy
- Class Members

- All
- Functions
- Variables

- a
- b
- c
- d
- e
- f
- g
- h
- i
- k
- l
- m
- n
- o
- p
- r
- s
- t
- u
- v
- w
- ~

### - s -

- ScoreHQThreshold()
  : GeneticMaterial- ScoreReproduction()
    : GeneticMaterial- SendMessage()
      : Vole\_Population\_Manager- Set\_Age()
        : Vole\_Base- SetAllele()
          : GeneticMaterial
          , GeneticMaterial1616- SetAside()
            : SetAside- SetDirectFlag()
              : Vole\_Base
              , GeneticMaterial
              , GeneticMaterial1616- SetFarmNumber()
                : Farm- SetFertile()
                  : Vole\_Male- SetFile()
                    : probe\_data- SetGeneticFlag()
                      : GeneticMaterial1616
                      , Vole\_Base
                      , GeneticMaterial- SetLocation()
                        : Vole\_Base
                        , Vole\_Male
                        , Vole\_Female- Setm\_Mature()
                          : Vole\_Base- SetNoProbes()
                            : Population\_Manager- SetWeight()
                              : Vole\_Base- SetX()
                                : TAnimal- SetY()
                                  : TAnimal- Shuffle()
                                    : Population\_Manager- Shuffle\_or\_Sort()
                                      : Population\_Manager- SimpleEvent()
                                        : Crop- SleepAllDay()
                                          : Farm- SortState()
                                            : Population\_Manager- SortX()
                                              : Population\_Manager- SortXIndex()
                                                : Population\_Manager- SortY()
                                                  : Population\_Manager- SpeciesSpecificReporting()
                                                    : Population\_Manager- SpringHarrow()
                                                      : Farm- SpringPlough()
                                                        : Farm- SpringRoll()
                                                          : Farm- SpringSow()
                                                            : Farm- st\_BecomeReproductive()
                                                              : Vole\_Female- st\_Dispersal()
                                                                : TPredator- st\_Dying()
                                                                  : Vole\_Base- st\_Eval\_n\_Explore()
                                                                    : Vole\_Male- st\_Evaluate\_n\_Explore()
                                                                      : Vole\_Female- st\_GiveBirth()
                                                                        : Vole\_Female- st\_Hunting()
                                                                          : TPredator- st\_Infanticide()
                                                                            : Vole\_Male- st\_JuvenileExplore()
                                                                              : Vole\_Male- st\_Lactating()
                                                                                : Vole\_Female- st\_Mating()
                                                                                  : Vole\_Female- st\_Maturation()
                                                                                    : Vole\_Male- st\_Movement()
                                                                                      : TPredator- st\_ReproBehaviour()
                                                                                        : Vole\_Female- st\_Special\_Explore()
                                                                                          : Vole\_Female- st\_UpdateGestation()
                                                                                            : Vole\_Female- Step()
                                                                                              : Vole\_Base
                                                                                              , TALMaSSObject
                                                                                              , TAnimal
                                                                                              , TPredator
                                                                                              , Weasel
                                                                                              , Owl
                                                                                              , Vole\_Male
                                                                                              , Vole\_Female- StepFinished()
                                                                                                : TPredator\_Population\_Manager
                                                                                                , Population\_Manager- StrawChopping()
                                                                                                  : Farm- Strigling()
                                                                                                    : Farm- StriglingSow()
                                                                                                      : Farm- StubbleHarrowing()
                                                                                                        : Farm- Supply\_m\_Location\_x()
                                                                                                          : TAnimal- Supply\_m\_Location\_y()
                                                                                                            : TAnimal- supply\_no\_inds()
                                                                                                              : TPredator\_Population\_Manager- SupplyAge()
                                                                                                                : Vole\_Base- SupplyAllele()
                                                                                                                  : Vole\_Base- SupplyAN()
                                                                                                                    : AlleleFreq- SupplyCovPosx()
                                                                                                                      : Population\_Manager- SupplyCovPosy()
                                                                                                                        : Population\_Manager- SupplyFarmOwnerRef()
                                                                                                                          : TAnimal- SupplyGenes()
                                                                                                                            : Vole\_Base- SupplyGrowthStartDate()
                                                                                                                              : Vole\_Population\_Manager- SupplyHeteroZyg()
                                                                                                                                : Vole\_Base- SupplyHomoZyg()
                                                                                                                                  : Vole\_Base- SupplyHowManyVoles()
                                                                                                                                    : Vole\_Population\_Manager- SupplyInOlderTerr()
                                                                                                                                      : Vole\_Population\_Manager- SupplyListIndexSize()
                                                                                                                                        : Population\_Manager- SupplyListName()
                                                                                                                                          : Population\_Manager- SupplyListNameLength()
                                                                                                                                            : Population\_Manager- SupplyListSize()
                                                                                                                                              : Population\_Manager- SupplyLocXY()
                                                                                                                                                : Population\_Manager- SupplyNoOfYoung()
                                                                                                                                                  : Vole\_Female- SupplyOlderFemales()
                                                                                                                                                    : Vole\_Population\_Manager- SupplyPegPosx()
                                                                                                                                                      : Population\_Manager- SupplyPegPosy()
                                                                                                                                                        : Population\_Manager- SupplyPosition()
                                                                                                                                                          : TAnimal- SupplySex()
                                                                                                                                                            : Vole\_Base- SupplySimH()
                                                                                                                                                              : Population\_Manager- SupplySimW()
                                                                                                                                                                : Population\_Manager- SupplyState()
                                                                                                                                                                  : Population\_Manager- SupplyStateList()
                                                                                                                                                                    : Population\_Manager- SupplyStateNames()
                                                                                                                                                                      : Population\_Manager- SupplyStateNamesLength()
                                                                                                                                                                        : Population\_Manager- SupplyStepSize()
                                                                                                                                                                          : Population\_Manager- SupplyTerritorial()
                                                                                                                                                                            : Vole\_Base- SupplyVoleList()
                                                                                                                                                                              : Vole\_Population\_Manager- SupplyX()
                                                                                                                                                                                : Vole\_Base- SupplyY()
                                                                                                                                                                                  : Vole\_Base- Swathing()
                                                                                                                                                                                    : Farm- SynInsecticideTreat()
                                                                                                                                                                                      : Farm

---

Generated on Thu Jan 22 14:13:45 2009 for ALMaSS ODDox by 
 1.5.6
